# Incidence, Phenotypes, and Genotypes of Neonatal Diabetes: A 16-Year Experience. The Rare Genetic Etiologies of Neonatal Diabetes Are Common in Sudan

**DOI:** 10.1155/2024/2032425

**Published:** 2024-02-15

**Authors:** Samar S. Hassan, Salwa A. Musa, Elisa De Franco, Russel Donis Frew, Omer O. Babiker, Ghassan F. Mohamadsalih, Areej A. Ibrahim, Samar Abu Samra, Mohamed A. Abdullah

**Affiliations:** ^1^Department of Pediatric Endocrine and Diabetes, Gaafar Ibn Auf Pediatric Tertiary Hospital, Khartoum, Sudan; ^2^Sudan Childhood Diabetes Center, Khartoum, Sudan; ^3^Department of Pediatric and Child Health, Faculty of Medicine, AL-Neelain University, Khartoum, Sudan; ^4^Institute of Biomedical and Clinical Science, Faculty of Health and Life Sciences, University of Exeter, Exeter, UK; ^5^Department of Pediatrics, Faculty of Medicine, Omdurman Islamic University, Khartoum, Sudan; ^6^Division of Endocrine, Department of Pediatric Medicine, Sidra Hospital, Doha, Qatar; ^7^Division of Pediatric Endocrine, Department of Pediatrics, Prince Mohammed Bin Abdulaziz Hospital, Madinah, Saudi Arabia; ^8^Department of Pediatrics, Faculty of Medicine, University of Khartoum, Khartoum, Sudan

## Abstract

Neonatal diabetes (ND) is a rare subtype of diabetes occurring in the first 6 months of life. High incidence has been reported among populations with high rates of consanguineous marriage. However, there is paucity of reported data from sub-Saharan African countries. We report the incidence, genotype, and phenotype of ND in a large cohort from Sudan and compare these findings to regional and international data. All infants with onset of diabetes in the first 6 months of life, attending one of the only two tertiary pediatric diabetes centers in Sudan, Gaafar Ibn Auf Pediatric Tertiary Hospital and Sudan Childhood Diabetes Center, during the period of January 2006 to December 2022 were included. Medical records were reviewed for demographic and clinical information. Genetic testing was performed for 48 patients by the Exeter Genomics laboratory in the UK and for one patient by the University of Cambridge, Metabolic Research Laboratories, UK. The estimated incidence was 4.8 per 100,000 live births. Forty-nine ND patients from 45 unrelated families were identified, and a genetic diagnosis was confirmed in 37 patients (75.5%) from 33 unrelated families. Consanguinity was reported in 34 families (75.6%). The commonest genetic cause for permanent neonatal diabetes was *EIF2AK3* recessive variants causing Wolcott–Rallison syndrome (18.92%). Pathogenic variants in two recently identified genes, *ZNF808* and *NARS2*, were found in three patients each (8.11%). Activating variants in *KCNJ11* and *ABCC8* were identified in four (10.81%) and two (5.41%) patients, respectively. Apart from hyperglycemia, the commonest clinical presentations included dehydration, failure to thrive, and diabetic ketoacidosis. ND in Sudan has a different pattern of etiologies compared to Western and Asian populations yet similar to some Arab countries with *EIF2AK3* mutations being the commonest cause. Pathogenic variants in recently identified genes reflect the impact of genome sequencing on increasing the rate of genetic diagnosis.

## 1. Introduction

Neonatal diabetes (ND) is a rare cause of diabetes characterized by disease onset in the first 6 months of life. It is clinically heterogeneous, with approximately half of the patients experiencing diabetes remission and relapse later in life, a subtype known as transient neonatal diabetes (TND) [1]. The remainder of the patients either have isolated permanent neonatal diabetes (IPND) or syndromic subtypes of neonatal diabetes (SND), the later often characterized by diabetes as the presenting feature before development of additional extra-pancreatic manifestations. The reported incidence of ND varies between different countries and the estimated incidence in Western countries is 1 in 100–500,000 live births [2, 3]. However, recent studies from Arab countries have reported incidences as high as 2.2 and 4.7 per 100,000 from Oman and Kingdom of Saudi Arabia (KSA), respectively [4, 5]. In the last decade, the introduction of exome and genome sequencing has led to the identification of new genetic etiologies, significantly expanding our knowledge on the causes and clinical spectrum of ND. To date, over 30 genetic causes of ND have been confirmed [6]. In Western and Asian countries the commonest causes of permanent neonatal diabetes (PND) are activating pathogenic variants in the *KCNJ11* and *ABCC8* genes, which encode the Kir6.2 and SUR1 subunits of the beta cell ATP-sensitive potassium (KATP) channel [7]. However, patterns of genetic etiologies causing PND in Arab populations have been reported to be markedly different [8].

There are some published reports from sub-Saharan African (SSA) countries on the incidence of type 1 (T1DM) and type 2 diabetes mellitus (T2DM), but to the best of our knowledge, there have been no published reports on the incidence of the genetic causes of ND so far. Sudan is an African Arab country with a high rate of consanguineous marriages. The aims of this study were to describe the diversity of phenotypes and genotypes of ND in a large cohort of Sudanese patients and compare this to the previously reported regional and international data.

## 2. Methods

This was a retrospective study conducted in Gaafar Ibn Auf Pediatric Tertiary Hospital (GIA) and Sudan Childhood Diabetes Center (SCDC) where inpatient and outpatient services are provided for pediatric diabetic patients. These two centers are located in the state capital, Khartoum, and are considered the only tertiary diabetes centers in Sudan, receiving referral from all states in the country. All infants presenting with diabetes in the first six months of life between January 2006 and December 2022 were included in this study. Medical records were reviewed for demographic data including sex, age at onset, consanguinity, family history, and other clinical data. Additional information included insulin or sulfonyl urea therapies and the current medical status of patients.

Following consent, DNA was extracted from peripheral blood using standard methods from 49 patients clinically diagnosed with ND. Genetic testing for one patient was performed by the University of Cambridge, Institute of Metabolic Science, while the remaining 48 patients had genetic testing performed by Exeter Genomics Laboratory in the UK as previously described [9]. Briefly, initial rapid testing for pathogenic variants in the *KCNJ11, ABCC8*, and *INS* genes was performed by Sanger sequencing (primers available on request). For patients who had a clinical phenotype suggestive of a specific subtype, Sanger sequencing of specific genes was carried out (primer details available on request). In individuals without a pathogenic variant identified by the initial analysis and for whom the phenotype was not specific for any known ND subtype, sequencing of all known neonatal diabetes genes (full list available at https://www.diabetesgenes.org/tests-for-diabetes-subtypes/targeted-next-generation-sequencing-analysis-of-45-monogenic-diabetes-genes/) was performed by next generation sequencing [10]. Methylation-specific MLPA for the 6q24 transient neonatal diabetes region was carried out for patients who either had transient neonatal diabetes or were aged <12 months at testing. Genome sequencing was performed for five patients for whom a pathogenic variant in a known gene was not identified by previous analysis.

Sanger sequencing analysis of the coding sequence and splice junctions of the *INSR* gene for one patient was performed at Cambridge Metabolic Institute Science. In brief, PCR amplification of exons and flanking sequences was followed by purification by Exo-SAP-IT (Affymetrix, Santa Clara, CA). The samples were then sequenced using a Big Dye Terminator v3.1 cycle sequencing kit in an ABI3730 genetic analyzer (Applied Biosystems, Foster City, CA, USA) after cleaning using Agencourt AMPure XP Beads (Beckman Coulter Inc, Atlanta, GA, USA). Sequence data were analyzed using Sequencher (Gene Codes Corp., Ann Arbor, MI, USA).

Data were analyzed using SSPS software version 27. Descriptive analysis of quantitative data and frequencies was conducted of normally distributed categorical data, which were expressed as mean ± SD and range when not normally distributed. *P* value <0.05 is considered significant with 95% confidence interval. The incidence was calculated using the total number of patients diagnosed with ND between 2015 and 2019, compared to the total number of live births in Sudan over the same period of time. Data on the annual live birth rate were provided by the Central Bureau of statistics of Sudan. The most recent published studies on ND from regional and international countries were selected to compare with our data. The selection was based on MEDLINE (via PubMed), EMBASE (via Elsevier), and Google Scholar advanced search using Boolean operation, nesting, truncation and quotes for phrases and was filtered by year of publication. The study was approved by the ethics and research committee of both previously mentioned centers.

## 3. Results

### 3.1. Incidence of ND in Sudan

**Figure 1:**
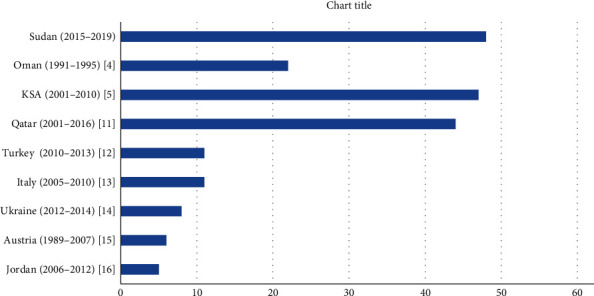
Incidence of neonatal diabetes among different regions (incidence 1 per 1,000,000 live births). References for other countries are given in square brackets.

Twenty-one individuals were diagnosed with ND between 2015 and 2019. From the total birth rate of 433,906 during this 5-year period, the calculated incidence of ND in Sudan was 1 in 20,833 live births (4.8 per 100,000 live births).  Figure 1 shows the incidence of ND from this study compared to those reported from other regional and international studies [4, 5, 11–16].

### 3.2. Demographical and Clinical Features of the Cohort

Patients' characteristics are summarized in Table 1. A total of 49 patients with ND from 45 unrelated families were included in the study; all were of Sudanese origin. The mean age of presentation was 59 days (range: 0–180), and the male to female ratio was 1.7 : 1.

Consanguinity (first- and second-degree cousins) was reported in 34 families (75.5%), and eight families (17.7%) reported history of early infant death of siblings with similar clinical features but no specific diagnosis. Apart from hyperglycemia, common clinical findings were mild to severe dehydration (42.8%) and failure to thrive (40.8%). Diabetic ketoacidosis (DKA) was the presenting sign in 20 patients (40.8%). Some patients showed extra-pancreatic features specific to the genetic etiology and included different clinical presentations.

### 3.3. Clinical Subtypes of Neonatal Diabetes and Molecular Genetic Studies

ND was clinically classified into three categories, TND (i.e., remission of diabetes by the age of 2 years), IPND (i.e., ND with no remission and no extra pancreatic clinical features), or SND (i.e., PND with extra pancreatic features). Remission was confirmed in five patients with TND, features suggestive of SND were found in 22 and the remaining 22 were PND.

**Figure 2:**
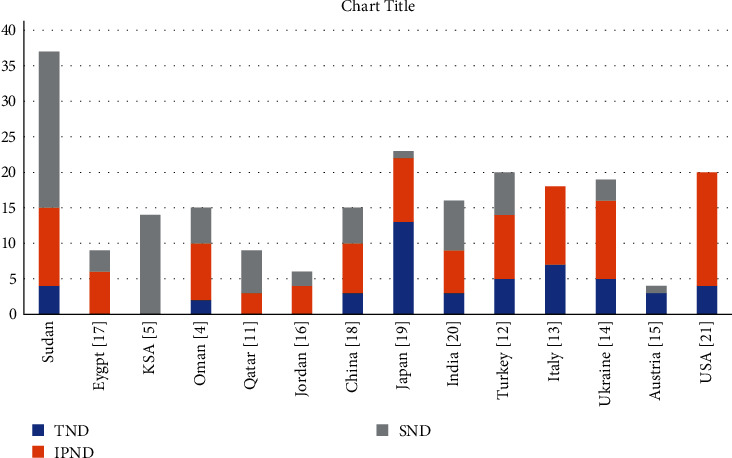
The numbers and subtypes of neonatal diabetes among different regions. References for other countries are given in square brackets.

Genetic testing for all known ND genes was performed until a causative variant was identified. Genome sequencing was performed for five patients for whom initial testing did not identify a likely genetic cause. A genetic diagnosis was confirmed for 37 probands (75.5%). Overall, variants in thirteen different genetic etiologies were identified in our cohort (Table 2). A genetic cause was confirmed for 4/5 TND, 22/22 SND, and 11/22 IPND patients. No likely genetic cause was identified for 12 individuals (11 with IPND and 1 with TND; Table 2). Our data on subtypes of ND were compared to the most recent studies conducted in regional and international countries as illustrated in Figure 2 [4, 5, 11–21].

#### 3.3.1. Transient Neonatal Diabetes (TND) (*n* = 5)

A genetic cause was confirmed in four out of five patients with TND (Table 2). Methylation abnormalities on chromosome 6q24 were identified in 3 of them, two were due to maternal hypomethylation and one was paternal uniparental disomy. A fourth patient with TND was homozygous for a *SLC2A2* pathogenic variant (p. Arg53Ter) causing Fanconi–Beckel syndrome (FBS) and presented with hepatomegaly and hypophosphatemic rickets secondary to renal tubular acidosis. In the fifth patient, diabetes remitted at the age of 4 months and relapsed at the age of 12 years. This individual was heterozygous for an *INS* variant (c.107T >G, p.Val36Gly) of uncertain significance (Table 2).

#### 3.3.2. Isolated Permanent Neonatal Diabetes (IPND) (*n* = 11)

IPND was mainly caused by pathogenic variants in the *KCNJ11* and *ABCC8* gene, which were identified in four (10.81%) and two (5.41%) cases, respectively (Table 2). All patients with KATP channel mutations were heterozygous except for one patient who was homozygous for a pathogenic *ABCC8* missense variant. None of these patients had neurological manifestations and DEND (developmental delay, epilepsy, and neonatal diabetes) and iDEND mutations were not detected in our cohort.

Other mutations causing IPND included three patients (8.11%) with *INS* gene mutations (two heterozygous and one homozygous) and two siblings (5.41%) with a homozygous *GCK* gene mutation (p. Asp73Leufs ^*∗*^10).

#### 3.3.3. Syndromic Neonatal Diabetes (SND) (*n* = 22)

Overall, the commonest genetic cause of ND in our cohort was recessive *EIF2AK3* variants causing Wolcott–Rallison syndrome (WRS) which were identified in seven unrelated patients (18.92%; Table 2). All seven were homozygous for different variants and were the offspring of consanguineous heterozygous carriers. Unresponsive renal hypertension was a dominant clinical feature in one patient who had a family history of a sibling's death with the same clinical features including resistant hypertension but for whom genetic testing was not possible.

A homozygous stop-gain variant (p.Asn770Ilefs ^*∗*^98) in the recently identified gene *ZNF808* was identified in three patients (8.11%) from two different unrelated families. All of the patients had ND with poor growth, one patient with chronic diarrhea and the two siblings with clinical features of malabsorption. Testing for stool elastase was not available.

In addition, a rare homozygous variant (p.Phe216Leu), in a recently identified gene, NARS2, was identified in three unrelated patients (8.11%). All probands presented in severe state of DKA, seizures and fluctuating hyperglycemia and hypoglycemia with insulin therapy. Seizures were generalized tonic–clonic in two patients while one patient had Salam attack seizures. All three patients had renal and liver dysfunction, and one patient had hypertension that might have been secondary to renal disease and required multiple antihypertensive therapy. Sadly, all three patients died soon after their presentation with severe sepsis and end organ failure.

Biallelic variants in the *SLC19A2* gene, causing Roger syndrome (RS) or thiamine-responsive megaloblastic anemia (TRMA), were detected in three patients (8.11%) from two unrelated families. One patient developed stroke and neurological manifestations (Table 2).

Recessive variants in *SLC2A2* mutations causing Fanconi Bickel syndrome were detected in three unrelated patients one of which had TND (previously mentioned in Section 3.3.1). Hepatomegaly was the cardinal sign and diabetes presented with fasting hypoglycemia and postprandial hyperglycemia.

Other rare mutations within the SND group included a homozygous *INSR* gene mutation which was identified in one patient who died at the age of 8 years with severe DKA and multiorgan failure. She required very high doses of daily insulin (100U). She presented with DKA in the first few weeks of life with clinical features consistent with Rabson Mendenhall syndrome (RMS). She harbored a homozygous mutation (p.Ser323Leu), while both parents were heterozygous carriers.

Homozygous variants in the *AGPAT2* gene causing congenital generalized lipodystrophy type 1 (CGL) or Berardinelli–Seip congenital lipodystrophy (BSCL) were found in two unrelated males. One patient had dyslipidemia and xanthoma.

A homozygous variant within the distal enhancer of the *PTF1A* gene was identified in one female proband who presented with diabetes, hepatomegaly, liver derangement, anemia, malabsorption, and multiple skeletal dysplasia. Stool elastase was not measured in the patient. Sadly, she died at the age of 9 years following sepsis and severe DKA.

#### 3.3.4. No Causative Variant Identified (*n* = 11)

A genetic cause was not identified in 11 patients, and in addition, one patient was heterozygous for an *INS* variant of uncertain significance and was considered with TND (Table 2). The type 1 diabetes genetic risk score was >50^th^ centile for two patients, suggesting a likely diagnosis of early onset type 1 diabetes. Whole genome sequencing has been performed for three of the remaining cases, but not likely, causative variant has been identified so far. Clinical features of TND and PND overlap, so without a genetic diagnosis, the classification of these patients would depend on clinical remission on follow-up. In this study, patients with no genetic cause identified and no evidence of extra pancreatic features who were lost to follow up were considered IPND and this was a limiting factor that may have underestimated TND and SND.

### 3.4. Management and Current Medical Status

All patients were treated with different regimens of subcutaneous insulin U-100, NPH twice daily or multiple daily injections (MDI) using basal and bolus, with an average dose of 0.3–0.5 U/kg/day except for two patients, one with a homozygous *INSR* variant and another with *AGPAT2* mutations who required higher doses (>2 U/kg/day). None of the cases were on continuous insulin infusion (CII) pumps due to it unaffordable expenses.

Attributed to the loss of follow-up of some patients, our local experience in transitioning from insulin to sulphonyl urea was small and based on only three probands (2 with *ABCC8* and 1 with a *KCNJ11* gene mutations). Inpatient transitioning protocol was used, serial measurements of serum blood glucose and C peptide levels were used for dose adjustment of sulfonyl urea. Transition was successful only in two patients with ABBC8.

In SND, in addition to insulin, other treatment modalities were offered according to the associated extra-pancreatic disease and included thiamine for patients with RS to help improve glycemic control and correct the TRMA, one alpha cacitriol and phosphate supplementation for patients with FBS to treat the hypophosphatemic rickets, supportive treatment for patients with WRS who develop episodes of acute liver and renal dysfunction, pancreatic enzyme therapy for the two siblings with ZNF808 to help improve intestinal absorption and metformin for the patient with INSR mutation to help improve sensitivity to insulin and reduce the associated metabolic effects of insulin resistance.

The major limiting factor of this study was the loss of follow-up in twenty-five patients (51%; Table 1). Death was reported in seven patients of which DKA followed by liver failure were the commonest causes. Data on chronic complications and prognosis was not available.

## 4. Discussion

This is the first and largest study reporting the clinical profile and genetic etiologies of ND from SSA. The calculated incidence was comparable to recent reports from some Arab countries [4, 5]. In this study, statistical data on live births per year could be underreported due to large numbers of home deliveries in Sudan, especially in rural areas. When considering this in addition to the high rates of consanguineous marriage in the population, the total number of patients with ND could be underestimated; hence, the incidence is expected to be higher.

Sudan is a country with diverse ethnicity and has over 200 tribes which are classified into three major groups Afro-Asiatic, Nilo-Saharan, and Niger–Congo [22]. Sudan has been reported as one of the countries with highest rates of consanguineous marriage with an overall rate of 44.2%–63.3% and a first cousin marriage rate of 44.2%–49.5% [23]. Consanguinity is widely practiced by different Sudanese tribes in order for each tribe to preserve their own inherited tribal customs and traditions. Parental consanguinity heavily influenced the pattern of genetic causes of ND where thirty-five (92%) probands with a confirmed genetic cause had a homozygous causative variant.

Currently, there are not enough data published regarding presenting signs and symptoms of ND. The classical clinical features seen in T1DM including polyuria and polydipsia are not the typical presentation for ND [24]. Stress hyperglycemia occurring in the neonatal period in sick babies may be very difficult to differentiate from ND especially in preterm babies where hyperglycemia may have a prolonged course [25]. Delay in recognition of early signs of diabetes in infants may likely result in the increase incidence of DKA [24]. This was a relatively frequent presenting feature in our patients, and reports from Egypt and India showed high rates of 65% and 60.7%, respectively [17, 20].

TND is estimated to represent ∼50–60% of ND [1]. TND was diagnosed in less than a one-sixth of the patients in our cohort. A possible yet partial explanation for this relatively small number was the fact that some patients were either too young or lost follow up at the time of publication, making it difficult to determine whether they would go to remission in the future. In a study from Japan, TNDM was seen in 41.9% of patients with 84.6% of them having methylation abnormalities at the 6q24 locus [19]. Although the numbers in our cohort were relatively small, methylation abnormalities at 6q24 mutations were the leading cause.

KATP channel mutations are the leading cause of PND worldwide [13, 14, 26]. While these variants were overall a small representation in our cohort, they were the most common cause of IPND. Recessively inherited loss of function mutation in *GCK* have been reported as a common cause of ND among some consanguineous populations like Oman [4]. Despite the fact that *GCK* is present in brain cells, neurological manifestations are not usually associated with loss of function variants in this gene, and to our knowledge, the patient in our cohort is only the second case with neurological clinical features observed [27].

SND is rare among patients from Western countries, yet it was found to be common in Sudan, with more than half of the patients with a genetic mutation identified in our cohort having SND [13, 21]. The most frequent genetic cause of ND in this cohort was in fact recessive pathogenic variants in the *EIF2AK3* gene causing Wolcott–Rallison syndrome (WRS). Our findings were relatively comparable to reports from some Arab countries [5, 28–31]. Patients with WRS tend to present, with liver and renal dysfunction and skeletal dysplasia usually being diagnosed after diabetes onset, as this was evident in more than half of our patients. Liver disease was the commonest presentation (85.7%) in a study comparing clinical presentation of WRS from different Arab countries, and this remains the commonest cause of mortality [31].

A homozygous protein-truncating mutation in a primate-specific gene, *ZNF808* gene, was recently reported as a cause of pancreatic agenesis characterized by ND and exocrine pancreatic insufficiency (two of the three patients from our cohort were included in the original study) [32]. In addition, a homozygous missense variant in another recently described novel gene, *NARS2*, was identified in three probands who presented with ND, seizures, and sepsis. Variants in this gene have previously been reported to cause a mitochondrial disease, and only recently one report described two siblings presenting with ND and being compound heterozygous for variants in *NARS2* [33, 34]. The precise clinical features of these recently identified ND genetic subtypes are still being defined. The identification of recently identified genetic causes highlights the genetic heterogeneity of ND in Sudan, raising the possibility that more novel genetic etiologies may be the cause of ND in some of the patients in this cohort.

Mutations in SLC19A2 causing TRMA had been reported in one patient in each study from Egypt, KSA, and China, and none of them had any neurological manifestations [5, 17, 18]. Similar to our finding, anemia has been reported to be megaloblastic or nonmegaloblastic (normocytic and microcytic), with some reported cases responding to iron supplementation [5, 35, 36]. These reports should prompt the modification of the terminology of TRMA to one with a wider range to include nonmegaloblastic types of anemia. There are some studies that reported improvement of hearing and glycemic control following thiamine replacement, but this was not the case in our cohort [36, 37].

Characterization of genetic causes has led to drastic modifications in the management of ND [38]. In a report from the United States of America (USA), 11 out of 14 patients with mutations in KCNJ11 were successfully transferred from insulin to sulfonyl urea (glyburide) therapy [21]. Previous studies have also shown the benefit of sulfonyl urea therapy in improving neurological symptoms in patients with iDEND and DEND [39, 40]. Although our local experience on transitioning patients to sulphonyl urea was very small and none of our patients had DEND syndromes, it still reflects the benefit of genetic testing on the management of ND and the improvement of life style sparing families the burden of daily insulin injections, nevertheless providing the ability to counsel them on probabilities of future off springs. Genetic analysis also aided us in counseling patients with chr6. mutations on the expected remission of their diabetes and the possible relapse later in life.

The identification of genetic causes of ND has undoubtedly shed more light on the insight of pathogenesis and has also been influential on the management of SND allowing physician to anticipate and treat the associated extra pancreatic complications and predict the expected life expectancies [41]. In our cohort, this was reflected in the management of patients with RS, FBS, WRS, INSR, and ZNF808 gene mutations. Another successful impact of genetic analysis on management of SND is the use of Leptin in patients with CGL, which unfortunately was not available for patients in our cohort [42].

From another standpoint, management of patients with rare types of ND can be extremely challenging, for example, the management of DKA with large insulin doses in cases of INSR gene mutations [43, 44]. Our patient required ≥0.5 to 0.6 U/kg/hour, and sadly, she died at the age of 8 years with severe DKA and multiple end organ failure. We emphasize on the need of international guidelines for management of DKA in rare subtypes of ND. As the loss to follow-up of patients was a major limiting factor, the assessment of prognosis and long-term complications were beyond the scope of this study, and we highly recommend long-term studies to target the prognosis, life expectancy, and chronic complications of ND.

## 5. Conclusion

We report a large cohort study in ND from a country with high rates of consanguineous marriage where syndromic causes were common. The population of Sudan has a different genetic spectrum compared to Western and Asian populations, with recessive genetic causes being common. These included variants in *EIF2AK3*, which were the most common cause, in addition to homozygous variants in two recently reported etiological genes, *ZNF808* and *NARS2*. Further research on genetic causes of ND, especially among populations with high rates of consanguinity, may expand the spectrum of genetic causes and give further insights into the pathogenesis of this disease.

## Figures and Tables

**Table 1:** Patients' characteristics (*N* = 49).

Patients' characteristics	Frequency	Percentage (%)
Genetic mutation confirmed	37	75.5
No genetic mutation	12 (In one patient, a mutation of uncertain significance was identified)	24.5
Male : female	1.7 : 1	—
Mean age of onset (in days)	59	—
Families with consanguinity	34	75.5
Families with death of undiagnosed sibling	8	17.7
DKA at presentation	20	40.8
Clinical features		
Dehydration	21	42.9
Failure to thrive	20	40.8
Hepatomegaly/liver disorder	10	20.4
Skeletal dysplasia/rickets	7	14.3
Anemia/megaloblastic anemia	6	12.2
Acute kidney injury/renal disorder	6	12.2
Neurological disorder	6	12.2
Malabsorption/chronic diarrhea	4	8.2
Cardiac disorder	3	6.1
Polyuria/polydipsia	3	6.1
Current medical status		
On insulin	14	28.6
Remission off treatment	1	2
On sulfonyl urea	2	4.1
Lost to follow-up	25	51
Died	7	14.3
Total number	49	100

**Table 2:** Types of neonatal diabetes, clinical presentation, and genetic mutations.

Type of ND	Genetic mutation	Number of patients	Average age in days	Clinical features	Zygosity	Genetic description
TND	6q24	3	24	IUGR, FTT, Dehy, DKA	—	Maternal methylation defects paternal deletion
	*INS*	1	14	FTT, Deyh, DKA	Heterozygous	Variant of uncertain significance:
	*NM_00207*					c.107T >G, p.Val36Gly
	*SLC2A2*	1	45	Hepatomegaly, hypo, HPR, RTA	Homozygous	c.157C >T, p.Arg53 ^*∗*^
	NM00340					
IPND	*KCNJ11*	4	73	FTT, Dehy, DKA	Heterozygous	c.1000G >C, p.Gly334Arg
	NM_000525					c.158G >A, p.Gly53Asp
						c.175G >A, p.Val59Met
						c.175G >A, p.Val59Met
	*ABCC8*	2	58	FTT, Dehy	Homozygous	c.3940C >A,p.Arg1314Ser
	NM_0012871 74				Heterozygous	c.2476C >T, p.Arg826Trp
	*INS*	3	12	FTT, Dehy, DKA	Heterozygous	c.94G >A, p. Gly32Ser
	NM_00207				Heterozygous	c.188-31G >A, p.?
					Homozygous	c.-331C >G, p.?
	*GCK*	2 Sibs	42	Hemiparesis, seizure	Homozygous	c.216_217dup, p.Asp73fs
	NM_000162					
SND	*EIF2AK3*	7	42	SKD, LD	Homozygous	c.802_803dup, p.Pro269fs
	NM_004836					c.1912C >T, p.Arg638 ^*∗*^
						c.1739del, p.Asn581fs
						c.2967T >A, p.Tyr989 ^*∗*^
						c.3026C >T, p.Ser1009Phe
						c.1909C >T/p.Arg637 ^*∗*^
						c.1647 + 2T >A, p.?
	*SLC2A2*	2	53	Hepatomegaly, fasting hypo, HPR, RTA	Homozygous	c.157C >T, p.Arg53Ter
	NM00340					c.1171-2A >G, p.?
	*SLC19A2*	3 (2 sibs)	170	SNHL, anemia, CHD, DKA	Homozygous	c.327_334del, p. Ile109fs
	NM_006996					c.327_334del, p. Ile109fs
						c.2309del, p.Asn770fs
	*ZNF808*	3 (2 sibs)	53	Malabsorption, growth failure, DKA	Homozygous	p.Asn770Ilefs ^*∗*^98
	NM_0013214 25					
	*NARS2*	3	55	Seizure, HTN, sepsis, DKA	Homozygous	c.648C >G, p.Phe216Leu
	NM_024678					
	*APGAT2*	2	90	Generalized subcutaneous tissue loss, muscle hypertrophy, xanthoma	Homozygous	c.589-2A >G, p.?
	NM_006412					c.524G >C p.Arg175Pro
	*INSR*	1	40	Dysmorphism, IUGR, acanthosis nigricans, multiple ovarian cyst	Homozygous	p.Ser323Leu
	NM_000208					
	*PTF1A*	1	30	SKD, hepatomegaly, chronic diarrhea	Homozygous	Chr10:g.23508363A >G, p.?
	NM_178161					
No mutation	—	11	80	FTT, Dehy	—	—

ND, neonatal diabetes; TND, transient neonatal diabetes; IPND, isolated permanent neonatal diabetes; SND, syndromic neonatal diabetes; IUGR, intrauterine growth retardation; FTT, failure to thrive; DKA, diabetic ketoacidosis; Dehy, dehydration; HPR, hypophosphatemic rickets; RTA, renal tubular acidosis; hypo, hypoglycemia; SKD, skeletal dysplasia; LD, liver disorder; SNHL, sensory neural hearing loss; HTN, hypertension; CHD, congenital heart disease.

## Data Availability

Data are available on request. Contact corresponding author for availability.

## Abbreviations

BSCL: Berardinelli–Seip congenital lipodystrophy
CGL: Congenital generalized lipodystrophy type 1
CII: Continuous insulin infusion
DEND: Developmental delay, epilepsy, and neonatal diabetes
DKA: Diabetic ketoacidosis
FBS: Fanconi–Beckel syndrome
GIA: Gaafar Ibn Auf Pediatric Tertiary Hospital
IPND: Isolated permanent neonatal diabetes
KAT*P*: ATP-sensitive potassium channel
KSA: Kingdom of Saudi Arabia
MDI: Multiple daily injections
ND: Neonatal diabetes
PND: Permanent neonatal diabetes
RMS: Rabson–Mendenhall syndrome
RS: Roger syndrome
SCDC: Sudan Childhood Diabetes Center
SND: Syndromic subtypes of neonatal diabetes
SSA: Sub-Saharan African countries
T1DM: Type 1 diabetes mellitus
T2DM: Type 2 diabetes mellitus
TND: Transient neonatal diabetes
TRMA: Thiamine responsive megaloblastic anemia
USA: United States of America
WRS: Wolcott–Rallison syndrome.
